# Identification of required capabilities of digital health ecosystems when preventing and managing non-communicable diseases

**DOI:** 10.1177/20552076241271807

**Published:** 2024-09-12

**Authors:** Minna Pikkarainen, Marika Iivari, Julius F Gomes, Jouni Kaartinen, Yueqiang Xu, He Hong-Gu, Parisa Gazerani

**Affiliations:** 1 Department of Rehabilitation and Health Technology and Product Design, 60499 Oslo Metropolitan University, Oslo, Norway; 2 Martti Ahtisaari Institute, Oulu Business School, 6370 University of Oulu, Oulu, Finland; 3 Health data analytics, 3259 VTT Technical Research Centre of Finland, Espoo, Finland; 4 Empirical Software Engineering on Software, Systems, and Services, Faculty of Information Technology and Electrical Engineering, 6370 University of Oulu, Oulu, Finland; 5 Alice Lee Centre for Nursing Studies, Yong Loo Lin School of Medicine, 37580 National University of Singapore, Singapore, Singapore; 6 Department of Life Sciences and Health, 158935 Faculty of Health Sciences, Oslo Metropolitan University, Oslo, Norway; 7 Centre for Intelligent Musculoskeletal Health (CIM), 158935 Faculty of Health Sciences, Oslo Metropolitan University, Oslo, Norway; 8 Department of Health Science and Technology, Faculty of Medicine, 1004 Aalborg University, Aalborg, Denmark

**Keywords:** Digital health, capability, chronic diseases, complex systems theory, preventive, innovative

## Abstract

**Objective:**

Non-communicable diseases cause annual mortality for 41 million people worldwide. These diseases include coronary heart disease, cancer, stroke, diabetes, and musculoskeletal as well as mental disorders. Innovation ecosystems in healthcare are multifactor networks in which different stakeholders interact together to create socio-economic (patient and cost) value via research, co-creation, and traditional market activities. Although there is much evidence about the impact of digital health interventions and the capabilities needed to support individual actors and specific diseases in non-communicable disease prevention and management, the current understanding of the concept of innovation ecosystems associated with theories is not well understood. There is also a lack of research about innovation ecosystems in the healthcare context. Or understanding of the holistic perspective of the capabilities needed in innovation ecosystems to support future digital health. The objective of this study was to answer this research gap by identifying what capabilities are needed in future digital health ecosystems related to people with non-communicable diseases or at risk of non-communicable diseases. By doing this, the study will help different organisations and policies address this very challenging situation.

**Methods:**

To answer this objective, a qualitative interview-based study including 34 semi-structured interviews was conducted in Finland. Complex adaptive systems theory was used as a theoretical lens to analyse empirical data.

**Results and conclusion:**

Several new capabilities were identified for digital health innovation ecosystems to make organisation managers and policymakers aware of how to deal with future health system demands. From the organisational perspective, capabilities are needed to use non-medical and heterogeneous data to support better treatments and clinical decision-making and provide better and safer data access. From the management perspective, hospitals need capabilities to allow critical experts to participate in innovation work, and overall, all ecosystem actors need capabilities to orchestrate research and innovation actions in the area of digital health.

## Introduction

Non-communicable diseases (NCDs) are reported to cause annual mortality for 41 million people worldwide. These diseases include coronary heart disease, cancer, stroke, diabetes, and musculoskeletal as well as mental disorders.^1–4^ Implementation of digital health technologies (e.g. integrated management of information technology in health, medical images, electronic medical records, development of portable, mobile devices in health, access to e-health, telemedicine, and privacy of medical data) seems to have the potential for mitigation and elimination of existing healthcare challenges related to the non-communicable diseases, reducing costs, and helping healthcare providers in addition to improving user experience and enhancing the quality of care.^5^

The digitalisation of the healthcare sector has, in particular, been considered to respond to match cost increases due to the increase in non-communicable diseases and the demographic changes strongly linked to these.^5^ For instance, there is evidence that digital health interventions are effective in supporting patients with chronic conditions and have helped patients lower their pain and functional disability^.6^ There are many examples from different areas of non-communicable diseases where digital health interventions have proven to have positive potential in reducing the burden of chronic conditions at different levels of analysis: individuals, the health care system, and society.^6^

For instance, in musculoskeletal health (e.g. lower back pain and osteoarthritis), current evidence supports the efficiency of digital health interventions, especially in pain management and functional disability.^6^ In the case of cancer, digital health interventions have proved efficient concerning self-management support, telemonitoring, and health education.^7^ In the case of stroke, behaviour interventions are helpful, especially in supporting physical activity and self-monitoring.^8,9^ In the case of mental health, tailored digital interventions have proved to have promising results in terms of presenteeism, stress levels, and sleep.^9^

However, digital health technologies and interventions alone are insufficient to address healthcare needs and demands at systems and societal levels. Therefore, digital health as a research phenomenon would benefit from a holistic ecosystems approach that simultaneously acknowledges both individual and system-level needs concerning technology. The concept of ‘digital health innovation ecosystem’ refers to a network of digital health communities consisting of interconnected, interrelated and interdependent digital health species, including healthcare stakeholders, healthcare institutions and digital health care devices situated in a digital health environment, who adopt the best-demonstrated practices that have been proven to be successful, and implementation of those practices through the use of information and communication technologies to monitor and improve the wellbeing and health of patients, to empower patients in the management of their health and that of their families.^10,11^ This concept is highly relevant for non-communicative disease conditions, especially for those that often accompany comorbid physical (e.g. pain and functional disability) and psychological (e.g. mental health and depression) conditions. Current literature on innovation ecosystems focuses on interactions between different actors such as service providers, firms, and research and education institutions.^12^ At the moment, there is incoherence in the definition of ‘innovation ecosystems’ in the literature. Hence, in this article, we focus on the multi-actor perspective of innovation ecosystems,^13^ where different stakeholders, that is, agents, interact together to create socio-economic (patient and cost) value via research, co-creation and traditional market activities.^12^ Although one can find much research related to innovation ecosystems,^14^ understanding innovation ecosystems and associated current theories is not fully addressed in academic research.^14^

There is also limited research on innovation ecosystems in the healthcare context. Extant research is related to the innovation ecosystems focusing on self-management and self-tracking apps, gamification, health and wellness apps, wireless sensors, health data exchange, health information and technology, and interoperability.^15^ Existing research is more focused on the innovation aspects of digital health ecosystems than a holistic perspective of the capabilities needed in innovation ecosystems to support future digital health. The complexity of interactions in digital healthcare ecosystems brings attention to how organisations can manage and operate in this kind of environment. Accordingly, a deeper exploration and understanding of the capabilities needed to foster digital health in the context of non-communicable diseases is required.

The capabilities addressed in the existing literature are, for instance, capabilities needed when developing appropriate information management processes, including the sense-making, collection, organisation, and dissemination of generated information, which are done using data analytics in digital health. There is also a need for the capability to install desired information behaviours and values (e.g. proactiveness, sharing, integrity) that are central, for example, to change the behaviour of individuals in order to decrease risks for chronic diseases, which in digital health are done using data analytics.^16,17^ Additionally, several leadership capabilities are required to orchestrate organisational capabilities and leverage the necessary resources needed to build organisational capabilities.^17^ In the healthcare field, the lack of resources is already one of the biggest problems, which is both hindering and speeding up digitalisation. However, in the current literature^16,17^ no meta-analysis has been conducted to reveal the capabilities needed to prevent and manage chronic disease conditions. As the context of digital health is complex, the heterogeneity of content, characteristics, and delivery of digital health interventions or the target population in the available studies for the review^6^ makes such meta-analysis challenging.

Complex adaptive systems (CAS) theory is an interdisciplinary framework used to understand systems that are composed of multiple interconnected components, which adapt and evolve in response to interactions within the system and with the external environment.^18^ It has been argued that ecosystem research would benefit from adopting better-founded theorising behind the core methodological choices. In particular, it has been suggested that CAS would be a useful approach when addressing ecosystem-related topics.^19^ Digital health interventions and digital health innovation ecosystems are complex activities due to the complex technological issues and organisational factors, which are often beyond the control of the health technology providers. In the case of digital health, the complexity is multiplied due to the need to combine different technological solutions, ethical and safety issues, work over the organisation’s borders, and in global companies.^20^ According to CAS theory, there is a mutual-influence relationship between the environment and agents.^21^ In this study, the environment is the digital health innovation ecosystem in which the agents, that is, health technology companies and healthcare provider organisations, operate to provide better health services for individuals. Digital health services are offered through the healthcare systems that include all organisations, people, and activities needed to provide well-being and healthcare for the people.^22^

Thus, understanding the key capabilities needed in digital health innovation ecosystems and their theoretical examination through CAS theory is still missing in designing or improving digital patient-centred health care for non-communicable diseases. Considering the research gaps and needs that also impact systems and society, we designed this qualitative interview study, in which 34 digital health experts were interviewed to address the following research question: What capabilities are needed in future digital health ecosystems (1) to bring value to individuals with non-communicable diseases or at risk of non-communicable diseases, (2) to orchestrate ecosystems and leverage resources and (3) to integrate solutions with operative systems and processes.

## Theoretical background for complex systems theory in digital health

CAS theory is an interdisciplinary framework used to understand systems that are composed of multiple interconnected components, which adapt and evolve in response to interactions within the system and with the external environment. Key characteristics of CAS^23^ include: (1) Multiple Agents: CAS are made up of numerous interacting agents (individuals, groups, organisations) that follow simple rules and make decisions based on local information. (2) Adaptation and Learning: Agents within CAS are capable of adapting their behaviours based on past experiences and interactions. This learning can lead to changes in the overall system dynamics over time. (3) Emergence: Complex behaviours and patterns emerge from the interactions among agents. These emergent properties are often unpredictable and cannot be understood by analyzing individual components alone. (4) Nonlinearity: Interactions in CAS are nonlinear, meaning small changes can have large effects, and the relationship between cause and effect is not straightforward. (5) Feedback Loops: CAS exhibit both positive and negative feedback loops, which can amplify or dampen changes, leading to complex dynamics and sometimes unexpected outcomes. (6) Self-Organisation: CAS can self-organise, meaning they can develop structure and order without central control. This self-organisation often arises from local interactions among agents. (7) Dynamism: CAS are dynamic and constantly evolving in response to internal and external pressures and (8) The environment, is identified by agents in the external population and connections between this external population and the focal population. It means that agents interact with (e.g. landscape and social context).

In healthcare, CAS theory has been used to understand how different components (patients, healthcare providers, institutions, and policies) interact and adapt to one another. Using CAS theory to understand future capabilities in digital health is also highly justified because digital health ecosystems are composed of numerous interconnected components, including patients, healthcare providers, technology platforms, data systems, and regulatory bodies. CAS theory helps to understand how these diverse agents interact and influence each other, highlighting the interdependencies that shape health outcomes and system performance.^24,25^ Additionally, healthcare ecosystems and digital health technologies continuously evolve in response to new information, innovations, and external pressures.^24^ CAS theory emphasises the adaptive nature of agents within a system. This perspective is crucial for understanding how digital health solutions can adapt over time, learn from data, and improve through feedback mechanisms.^26^ Furthermore, In digital health, complex behaviours and patterns often emerge from the interactions of various stakeholders and technologies. CAS theory provides tools to study these emergent properties, which are not predictable from the behaviour of individual components alone.^27^ Finally, CAS theory promotes a holistic view, considering the entire ecosystem rather than isolated components. This perspective is crucial for digital health, where the integration of technologies, policies, and human factors is essential for successful implementation and sustainability.^28^ In summary, CAS theory provides a robust framework for understanding and managing systems characterised by multiple interacting agents, adaptation, emergence, and dynamic evolution. It emphasises the importance of local interactions, feedback mechanisms, and the non-linear nature of complex systems.

**Figure 1.**
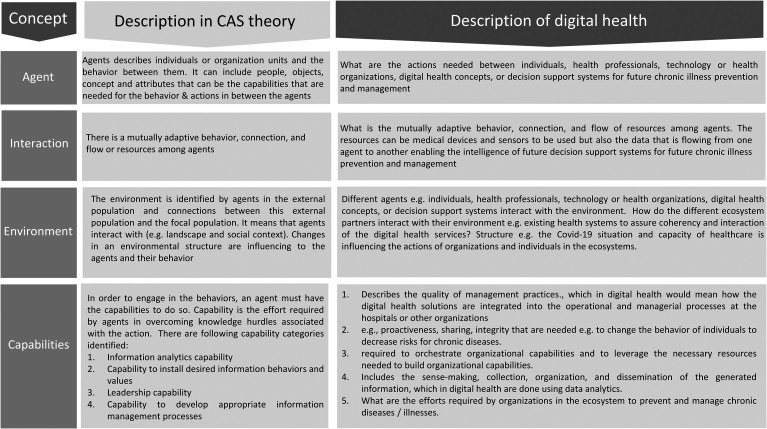
Mapping of complex adaptative system theory elements with digital health.

In this study, CAS theory was used as a theoretical lens to analyse empirical data. Digital health services could be characterised as CAS that are made up of several agents that interact with each other. Figure 1 maps the CAS theory elements onto digital health. The key elements discussed in detail in this article are agents, interaction, environment, and capabilities.

## Methods

### Study design

We chose an exploratory, qualitative research approach with semi-structured interviews and a thematic analysis.^29^ A qualitative approach and thematic analysis were chosen because they allow information to be collected directly from those experiencing the phenomenon under investigation.^30^ In this study, these people consisted of healthcare professionals, company representatives, and research organisation representatives. The design of this study can also characterised as phenomenology^31^ since the experiences of the participants were also determined from their capabilities.

**Figure 2.**
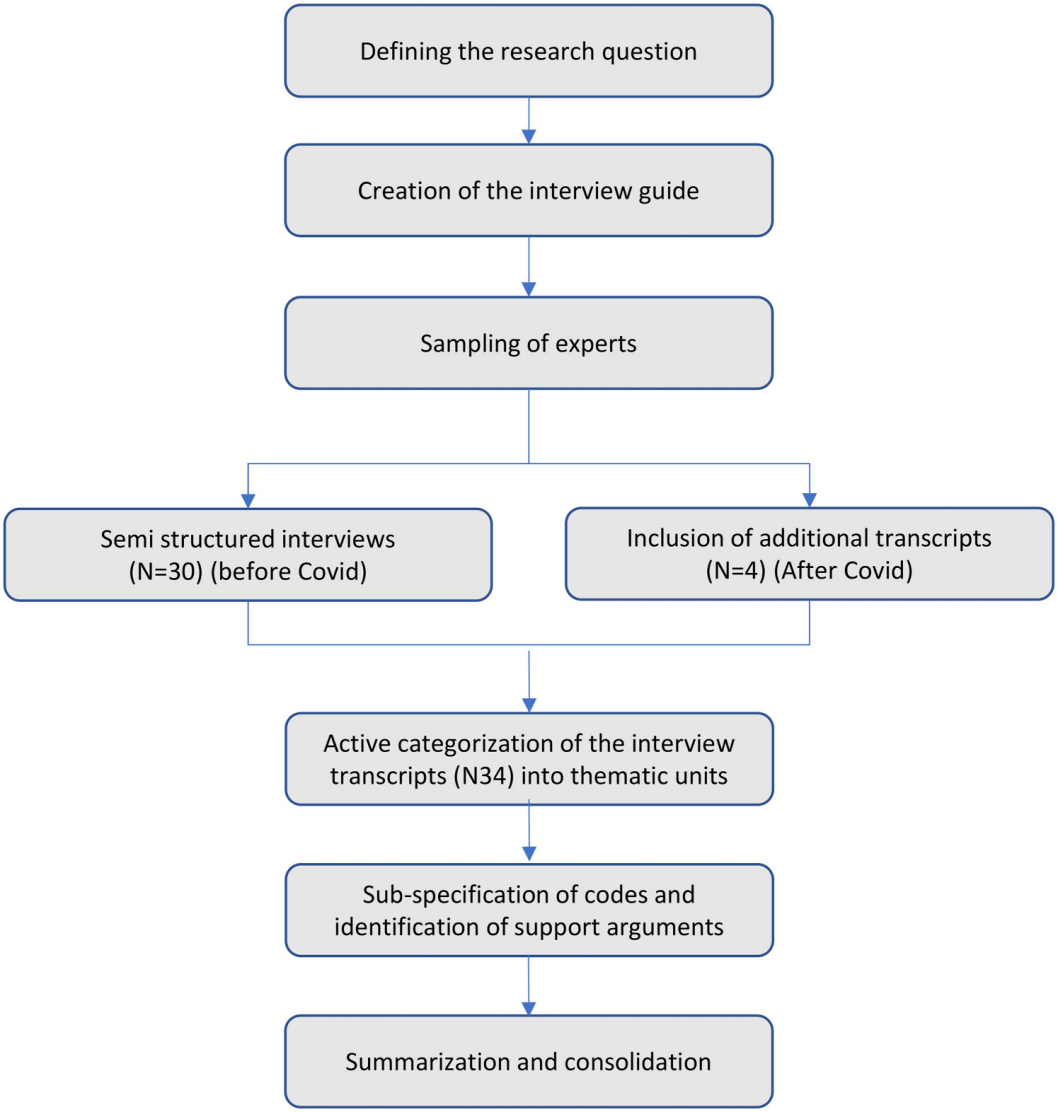
Research approach of the study.

The research approach of this study is described in Figure 2.

### Ethical issues

Ethical permission was not required for this type of study, but in order to follow the European General Data Protection Regulations (GDPR), specific informed-research-consent forms were prepared and signed by the study participants to ensure consent for the data usage in the conducted research.

### Study criteria

Three criteria were identified for participant's selection: (1) The participant is working in the digital health & non-communicable disease field and having more than 5 years’ experience working in a management position in this field. (2) Participant is a person who is really influencing the future directions of the digital health field, for example, he/she is involved in the strategy processes or creating new funding or funding programmes affecting the digital health future directions, and (3) Participants have a holistic understanding of the similarities among the different non-communicable disease areas.

### Sampling strategy

The 34 respondents fulfilling the identified criteria were identified for the study using purposive sampling. Due to the complex phenomena under investigation, maximum variation sampling, that is, heterogeneous sampling, was used to capture the broadest range of perspectives possible. This was done in order to ensure the maximum variation of researchers, managers, company representatives, and health professionals identified as key experts in digital health development in the area of non-communicable diseases. The selected sample in the study helped researchers to examine a subject from different angles, identifying important common patterns that are true across variations.^32^ Thus, the people who were interviewed for the study were working on many non-communicative diseases.

**Figure 3.**
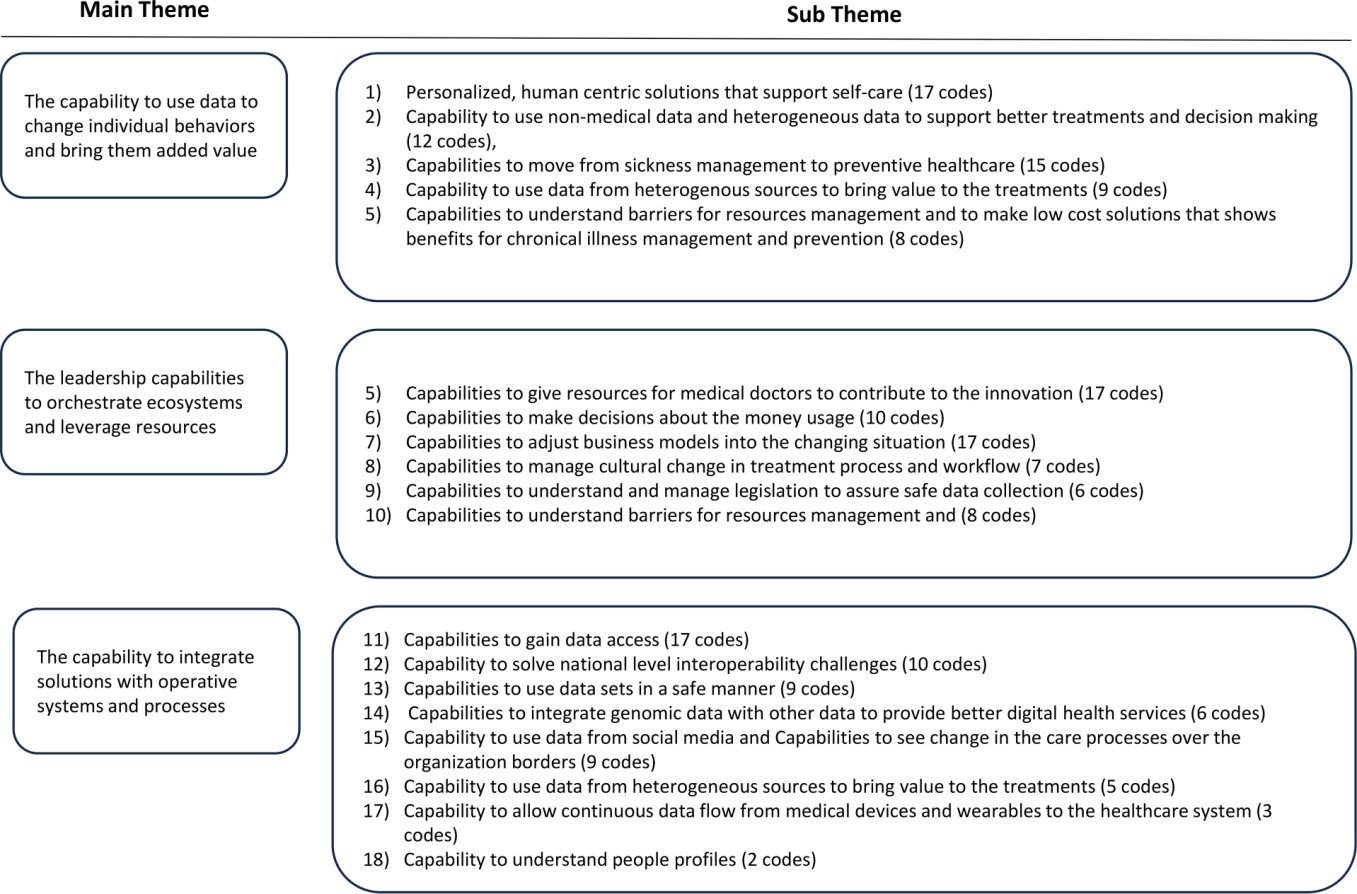
Analysis themes of the study.

The participants included 12 professors, senior researchers/medical experts who were mainly working on research related to new digital health technologies and technology innovations, eight company directors who are in charge of digital health technology-related R&D activities, and 14 directors of universities and research centres who were working with digital health and digital health-related research agendas as a part of their daily work activities. The directors in the universities and research organisations were involved in a more diverse area of digital health, covering several aspects of it as part of their work. Most of the interviewed experts from companies and research institutes were working in the domain of health systems and health data. In contrast, some experts were more specialised in rehabilitation, surgical care, e-health, brain health, and preventive healthcare in the different areas of non-communicable diseases (Figure 3). Based on this, we believe that the selected sample in the study is highly representative of experts and is among the key players involved in directing the future of digital healthcare.

The list of potential interviewees was carefully selected, discussed, and decided with the research team. All identified people were contacted first by email and then approached through telephone or arranged face-to-face meetings. The reasoning for the interest of the research theme was reported to the study participants. Some of the people interviewed were unfamiliar to the research team. Some of the interviewees’ research team members knew previously through various engagements in the digital health domain. Since we wanted to understand the phenomenon of digital health and its future directions from different perspectives, we involved people with varying areas of expertise in our study, that is, medical experts, industrial experts, and researchers, as explained earlier in this chapter.

All identified experts confirmed their participation in the study.

### Data collection

Interviews took place between 2019 and 2021, with 30 face-to-face interviews conducted before COVID-19 restrictions and four additional online interviews after restrictions were imposed. 1–2 interviewers and one interviewee were present in each interview, which ensured data triangulation and increased the validity of the selected qualitative research approach.^33^

The interviews aimed to build an understanding of the capabilities needed for future digital health for people living with chronic diseases or at risk of chronic diseases. The interview guideline was designed and validated together with the interview team, and the questions were related to defining digital health, the future of digital health, who benefits from digital health, and in which way they benefit from it (Appendix 1). The semi-structured interview framework enabled the interviewee's position, duties, and interests to lead the conversation. The interviews were conducted in English, lasting between 60 and 90 min. Field notes were made during the interviews by interviewers. After the interviews, notes were compared for data triangulation purposes. All the interviews were audio recorded and transcribed for analysis.

### Data analysis

Thus, we decided to use CAS theory as a theoretical lens to understand the required capabilities in complex, future digital health innovation ecosystems. The interview questions (Appendix 1) were purposefully general, looking at the future of digital health, focusing, for instance, on the benefits, hindrances, and future of digital health. The interview team reviewed and tested the questions before the interviews were started.

Capabilities in CAS theory were used as a theoretical lens to analyse the collected data. The capabilities were identified in the analysis phase through questions based on CAS theory: (1) What new capabilities are needed in digital health, especially considering actions between individuals, health professionals, technology and health organisations, digital health concepts, and decision support systems in health ecosystems? (2) What capabilities are needed for mutually adaptive behaviour and the connection and flow of resources between agents? (3) What capabilities are needed when different agents, for example, individuals, health professionals, technology or health organisations, or different elements such as digital health concepts or decision support systems interact with the environment and (4) How do the different ecosystem partners interact with their environment, for example, existing health systems to assure coherency and interaction of the digital health services) when the data were already collected. In our analysis, we used the inductive method for data sense-making as described by Glaser.^34^

The capabilities described by Park et al.^17^ were used as a starting point for the analysis, as their study provides a comprehensive contribution to the discussion on capabilities when looked through the CAS theory in the digital world. The analysis was done using Excel. The analysis identified new capabilities and linked them with the emerging categories, agents, and interactions. A thematic analysis approach^29^ was followed, and the categorisation framework developed by Grodal et al.^35^ was used for guidance. In the first step, we categorised the data into thematic units. In the second phase, codes were sub-specialised, refined, and stabilised by identifying arguments supporting the overarching theory until data saturation was achieved. In the final step, codes were summarised, consolidated, and discussed between 2 researchers. Based on the data, coding, and created coding tree, we revealed 20 new capabilities required for future digital health for people living with chronic diseases or at risk from chronic diseases in rapidly changing health ecosystems. It was also shown that individual centricity, data use, and an ecosystems approach played a significant role in the future digital health landscape. In total, we obtained 153 codes from the data. The results of the interviews were presented to a few of the interviewees in different seminars and events to validate the correctness of the analysis and collect feedback for the research results.

## Results

In the following sections, qualitative research data are mapped according to the theoretical framework of CAS theory. Qualitative data describing the capabilities was relevant when categorising the research results. We used the agent, interaction, and environment constructs of CAS theory. Our analysis focused on the capabilities needed to improve digital health (prevention and chronic disease management) between the agents (healthcare professionals, companies, and people) in a changing health ecosystem. This means that the capabilities are the key variable in our analysis.^36^

In Figure 3, we summarise the themes revealed from the data analysis related to CAS theory and the number of responses shown in the analysis.

### The capability to use data to change individual behaviours and bring them added value

Among the data-related factors, the participants (12/34) frequently viewed the capability to use non-medical data together with other heterogeneous data to support better treatments and clinical decision-making for people at risk of non-communicable diseases or those with non-communicable diseases. The data generated by various actors in the care processes need to be analysed using machine learning techniques. This is an enabler for improved treatments and decision-making among different ecosystem actors. For instance, one of the respondents (R1) mentioned:

I think that there could be more in the area where we are connecting (multi-modality) data together and processing that data further to gain a more holistic view and using tools like artificial intelligence (A1).

This is the case in acute stroke situations, for instance, where artificial intelligence (AI) and heterogeneous data usage could improve stroke assessment and decision-making.

Another category that emerged as potentially significant (14/34 answers) was the capability to move from managing chronic illnesses to preventive healthcare. It was revealed in 9/34 interviews that the emphasis on digital health should be placed on chronic illness risk prevention and pre- and post-stages of the care pathways. This is because these home care stages are the ones that bring the most return on investments related to future digital health. As stated by one of the respondents (R8),

We could start to move out of the care phase into the pre-and post-phase simply because that would probably get the fastest return on investment, increasing the load if we can avoid some of the illnesses or health issues and then in the post phase making sure that there is no relapse or return to the care phase.

This is the case in many non-communicable diseases, such as cardiovascular diseases, stroke, and musculoskeletal disorders. For instance, overwhelming evidence shows that lifestyle intervention programmes promoting healthy diets, physical activity, and modest body weight reductions could significantly prevent or delay the onset of type II diabetes among high-risk populations.

Increased capabilities were also needed in the digital health innovation ecosystem (17/34 interviews) to create more personalised, human-centric solutions for non-communicable disease prevention and monitoring that support self-care. This, however, requires the capability to provide easier access to health services, for example, in remote areas (6/34). As one of the respondents pointed out:

‘We should have technology that you can give individuals to provide personalised treatment in remote locations’ (R11). One key aspect revealed in one interview was the required capability ‘… to adopt a more comprehensive viewpoint for persons, patients or citizens’ (R5).

Capabilities are needed to develop citizen-centric, not technology-centric, services (1/34 answers). Additionally, the interviewees mentioned the capability to use citizen-collected data (2/34 answers) and help people treat themselves (1/34 answers).

From the hospital perspective, there was a need for greater capabilities to engage critical experts in innovation activities (16/34 interviews) to enable more meaningful innovation development. The results related to this proposition were divided into categories describing the capability to convince medical experts about the innovation work benefits (2/28), the capability to involve medical experts in discussing solution needs, the company's capability to build trust (2/28) when working with health professionals, the company ability to work with experts using an evidence-based approach, university capabilities to educate health experts about innovation perspectives, and the hospital management capability to manage costs and resources so participating in innovation work would be possible for medical experts. For instance, one of the respondents (R1) stated:

I still see the difficulty that companies, so enterprises coming in this medical field that, they don’t realise the … the realities of health systems which are, by discipline quite conservative … healthcare experts want to see evidence before they change everything.

### The leadership capabilities to orchestrate ecosystems and leverage resources

There is a need for different ecosystem players to develop the capabilities to orchestrate ecosystem-level innovation development for chronic disease prevention and support (16/34 interviews). According to the interviews, this would require capabilities to (1) create business models, (2) set common goals, (3) share ideas, (4) understand other stakeholder perspectives, (5) understand process complexity, (6) use resources at the ecosystem level, and (7) see the potential impact of future actions. For instance, (R6) stated:

It's an orchestration challenge concerning how you get them all together; it’s a very complicated systemic problem, and sometimes – even though you have a beautiful technical solution – you might not understand the customer aspect, or you may not have a proper means of design thinking, or you might not know the surrounding stakeholders very well.

The capability to understand and manage legislation was mentioned in 6/34 interviews. This is important for policymakers, public organisations, and companies. For example, one of the respondents (R7) argued that:

In Finland, we are in a good position in the sense that the legislation in terms of the secondary use of health information is already in place, so we have a system set by society, so we know how we can operate based on sensitive information that different data sources have about the population, and we understand the rules and how to use them.

The respondent points out that as a part of the national-level capability to manage and understand legislation, we need a more robust capability to carry out European-level actions to assure safe system interoperability globally. There was also a need for governmental capabilities to use and share money wisely between the different agents to support digitalisation activities (15/34 answers). This also concerns anticipating the long-term benefits of the decisions about different health outcomes. For example, (R3) mentioned that policymakers should have better capacities to create new programmes to support healthcare development:

The new Finnish government’s programme needs to do more to help the war against diabetes. I think it should be taken to a much higher level, and more resources need to be assigned.

The capabilities needed to provide data access were mentioned in 17/34 interviews. The data access capabilities were divided into the ability to gain access to data access from electronic health record systems and the ability to gain access to data through technological solutions, that is, specific algorithms, the use of AI, and automation. For instance, one of the respondents (R4) stated:

I think that with the help of artificial intelligence, for example, we can also then derive more personalised care for people, and I believe it could also result in better responses, saving people's money and time, also from the health professionals’ side so they can be more focused on the treatments that they prescribe to their patients.

It was found in the analysis (6/34 interviews) that there is a need to integrate genomic/biobank data with behavioural/sensor/wearable data that people generate daily. This helps to identify risk factors and to create better personalised digital health services. For instance, one respondent (R10) stated:

There are, of course driven by the habits of an individual, but also the genome, genetic profile of an individual. And that's something that you could get from the data, (R10).

The capability to understand and manage health systems as a whole was mentioned in 9/34 interviews. This was divided into the following categories: the capability to see and manage care processes across organisation borders, involve ecosystem actors in system-level solution development and understand the links between the social and policy systems. For instance, one respondent (R5) stated:

I also think that when developing these new healthcare solutions for citizen or patient purposes, we need to take into account the whole care system and the related policy system to scale up the new solutions we are developing.

### The capability to integrate solutions with operative systems and processes

Capabilities related to national-level interoperability were mentioned in 10/34 interviews. It was stated, for instance, that there is a need to manage the connectivity between different regional systems, manage the information flow between other systems, and use national platforms to support system interoperability. For instance, one of the respondents (R7) noted:

There are a huge number of information systems which are not very well interconnected, or it may not be possible to communicate between the different systems, or the coding of the information in the systems may not be coherent. Of course, this is one of the big chances that are currently happening, for instance, in Finland.

### Summary

As the above analysis shows, it is important for actors in the digital health ecosystem to increase the capabilities related to the availability of heterogeneous, medical, and non-medical data. This is needed to allow data usage for more personalised digital health interventions targeting more for the prevention of non-communicable diseases. From a holistic perspective, there is a need for both policymakers and health organisations to put more effort into the interoperability and data availability of digital healthcare solutions. Personalisation is needed in all non-communicable disease areas, whereas prevention is more emphasised in the areas where lifestyle risk factors have proven scientific evidence of the effect of lifestyle change on the decreased number of diseases in societies.

From the leadership perspective, key capabilities revealed from our analysis were to give resources for medical doctors to contribute to innovation, capabilities to make decisions about money usage, capabilities to adjust business models to the changing situation, capabilities to manage cultural change in the treatment process and workflow, capabilities to understand and manage legislation to assure safe data collection and capabilities to understand barriers for resources management. All the revealed leadership capabilities are related to the obstacles that are currently hindering innovation work and innovation implementation in digital health ecosystems. From the societal perspective, it was revealed that for the digital health ecosystems, it would be essential to improve the capabilities to gain data access and capability to solve national-level interoperability challenges and capabilities to use data sets safely.

## Discussion

Our study used the Complex Adaptative Systems theory as a theoretical lens to identify what capabilities are needed in future digital health ecosystems to change people’s behaviour and bring them added value, to lead and orchestrate ecosystems and resources, and to *integrate solutions with operative systems and processes.* In the case of increasing non-communicable diseases, especially healthcare personalisation and prevention emerged as central themes in digital health ecosystems.

### The capability to use data to change people’s behaviours and bring them added value

According^28–35^ to the interviews, it was particularly emphasised that the critical capabilities of the future of digital health should be more focused on the prevention that require personalisation of offered digital health interventions. However, according to Benis et al.,^28^ the opportunities to use electronic health data to manage personal health and share personal information would also require patient engagement, services, and trust in healthcare delivery.

Remote monitoring with digital tools has also been a research focus by several groups, including Brohman et al.,^37^ who studied a new telemonitoring initiative for empowering patients with chronic illness. In this system, a feedback ecosystem was implemented for telemonitoring.^37^ Recently, Yu et al.^38^ reported three cases representing the practicality and successful use of remote monitoring of senior citizens with the help of mobile health analytics.^38^ In the study of Yu et al.,^38^ individuals obtained precise assessments of the progression of existing chronic conditions or the risks of potential chronic diseases without clinical visits, improving their confidence in living independently. Thus, from an environmental perspective (in CAS theory), the patient-centred healthcare approach is growing rapidly.^39^ Our study highlights that there is a need to take the personal expectations of different users and ecosystem stakeholders better into account when developing and offering digital solutions, as has also been emphasised by Häikiö et al.^40^

A study from 2022^41^ has addressed some of the issues related to self-management strategies enabling patients with non-communicable diseases with the help of an online platform. However, in addition to our findings, patients who participated in this study^41^ identified limitations, including lower digital and health literacy. Therefore, barriers and enablers are gradually recognised to guide the gaps for further research. Other researchers have also contributed to shaping these critical areas for future research. Thompson et al.^42^ advanced the idea of temporal displacement of care (TDC). They provided evidence that healthcare value could be provided by strategic actions taken at specific time points during the treatment. This notion is highly relevant for chronic diseases, and the researchers in this study^42^ identified that earlier use of low-intervention treatments could reduce overall healthcare costs.

Our study also advances the current discussions in facing or planning to face chronic diseases with the help of digital health. In 2020, Jiang and Cameron^43^ proposed a self-monitoring strategy where patients with chronic diseases can use digital technology to manage their diseases and associated risk factors. They also claim that more efforts are needed to understand better the intervention components’ effects to assess impacts and design more effective interventions. This aligns with our analysis, which reveals that new capabilities are needed to develop more effective interventions and enable better data usage among different ecosystem agents. Data are specifically required when co-creating more personalised applications and components on smart devices (mobile apps), tracking programmes available via websites, wearable devices that can monitor and record various health parameters, and other devices built based on information technology for self-monitoring.

### The leadership capabilities to orchestrate ecosystems and leverage resources

Our analysis revealed that leadership and business modelling capabilities are needed in both healthcare provider organisations and companies that are developing AI technology to support professionals during the decision-making process. The core business opportunity in the ecosystems relates to the growing availability of health data collected through digital health.^44^ Business model discussions could help ecosystem agents understand different motives for why and how they can contribute to the ecosystem. Value creation for patients and healthcare experts is a key motivator for business modelling since the companies feel that more value created for end users will result in more value captured for the business.^44^

Leadership capabilities to orchestrate digital health innovation ecosystems and leverage resources into them have been more deeply discussed in the innovation management literature (e.g. Hurmelinna-Laukkanen et al.,^45^ Kemppainen et al.^46^). One way to ensure more resources to contribute to the innovation in healthcare provider organisations is the lead user approach,^45^ which allows the selection of core users from the healthcare organisations or the related networks to support innovation activities. As revealed in our study, there is also a need for improved capabilities to make better decisions on public health policies related to money usage and legislation on the use of data. These decisions made by policymakers are typically driven by various, continuously changing, and interlinked determinants that could be better managed using data-driven decision support tools.^47^

There are also specific laws and regulations for accountability associated with designing AI-based health systems that companies should have more capabilities to follow. For example, developing AI for specific health-related tasks and heterogeneous (medical and non-medical) data availability is essential to ensure that AI-driven outcomes and personalisation should not be done safely without causing harm to patients or society.^48^ Similarly, as in our analysis, Thompson et al.^42^ also emphasise the need for capabilities related to security mechanisms but more from the technological perspective, for instance, related to user authentication and intruder detection. Referring to the context of non-communicable disease prevention, they suggest that a stable IT infrastructure is needed to enable practitioners to build effective analytical capability that includes ‘push’ and ‘pull’ data capabilities, such as ‘pushing’ an alert when a patient has missed a prescription and ‘pulling’ updates when a patient gets immunisations or receives a therapy treatment at home.

In line with our study, Jiang and Cameron^43^ and Pikkarainen et al.^49^ suggest that more advanced data usage and analysis capabilities are critically needed. However, Jian and Cameron argue that future research should also compare the effectiveness of different feedback modes and formats to support self-monitoring and self-management aspects of digital interventions. At the same time, Pikkarainen et al.^49^ emphasise that from the platform organisation's perspective, there is also a need for capabilities to gain information about the processes, impact studies, gamification, the meaningfulness of new components and functionalities and the possibilities to develop analytics using hospital data.

In the ecosystem context, the actors also need more capabilities to properly handle privacy and security challenges between the agents (e.g. healthcare provider organisations and companies).^50^ However, the impact of the use and implications of these different technologies also require capabilities to have a more systematic approach, which we found in our study.

Similarly, as in our analysis, Thompson et al.^42^ also emphasise the need for security mechanisms related to user authentication and intruder detection capabilities. They suggest that a stable IT infrastructure is needed to enable practitioners to build effective analytical capability that includes ‘push’ and ‘pull’ data capabilities, such as ‘pushing’ an alert when a patient has missed a prescription and ‘pulling’ updates when a patient gets immunisations or receives treatment at home.^42^

### The capability to integrate solutions with operative systems and processes

Interoperability supports sharing health and illness experiences, coordinated care, and research for citizen empowerment and improved health outcomes. The adoption of interoperability principles needs to align with European and national regulations.^51^ Data generated within one agent in the ecosystem can be used differently by another agent in the ecosystem.^52^ Standardisation in medical informatics enables the interconnection and interoperability between care and research systems.^51^ Although much effort has already been put in Finland to improve local clinical systems and allow better alignment for easier data integration, it was revealed in our study that ecosystem actors would need better capabilities at regional and national levels.

To enable the prevention of non-communicable diseases, the capabilities are also needed to allow data access among other agents in the ecosystems (see also Pikkarainen et al.^49^), that is, communities, to ensure meaningful use of data and their applications for the advancement of healthcare delivery by partnering with community members to gather and synthesise data. For example, members can be included in churches, hairdressers, tribal areas, or community meetings. This could promote health equity through culturally appropriate solutions to ascertain health disparities, as it is noted in the literature that it is a considerable concern and a neglected domain.^53^

This goes beyond the results of Thompson et al.,^42^ who also remind us that related to preventive interventions such as diabetes solutions, data sharing always requires people’s willingness to share health data, protocols to master patient index through which longitudinal interventions and cost data from various providers are accurately combined and linked to each patient. Achieving health equity, that is, access to data and health resources through digital health applications, is challenging but offers an excellent opportunity for further research and investigation on implementing it in practice.

### Synthesis of the findings

Our results make a significant contribution to the field of digital health by bringing more insights into the holistic view of capabilities that are needed related to nonlinear interdependencies between key organisational capabilities and between the different ecosystem agents and their potential future effects on future digital health. Key takeaways and future capability priority suggestions are presented in Table 1. As extant research explores the capabilities from the perspective of individual actors in an ecosystem, they point out the need for capabilities related to the impact of digital health interventions, design, security aspects, and data access. This research brings more insights into what capabilities we need to support digital health ecosystems, focusing on personalisation, data usage, interoperability, business models, and ecosystem orchestration.

**Table 1.** Key takeaways and future capability priority suggestions.

**Already known capability needs**
Capabilities to understand how intermediate outcomes mediate the effects of digital health intervention use on chronic care goal achievement. Capabilities to understand how to design digital health interventions so that intervention satisfaction and compliance are improved. Capabilities related to security mechanisms but more from the technological perspective, for instance, related to user authentication and intruder detection Capabilities to enable data access and more advanced data usage and analysis in platform organisations
**Future research directions and capability needs from an ecosystem perspective**
Capabilities to achieve the personal expectations of different ecosystem stakeholders better into account when developing and offering digital solutions Capabilities to provide healthcare value to different ecosystem agents through strategic actions that are taken at specific time points during the treatment Capabilities are needed to enable better data usage among different ecosystem agents. Data are specifically required when co-creating more personalised applications and components Capabilities are needed to do business modelling to help ecosystem agents understand different motives on why and how they can give their contributions to the ecosystem Capabilities are needed at the policy level to make better decisions about money, data usage, and legislation related to future digital health interventions Capabilities are needed nationally and regionally to improve the interoperability of digital health interventions

New capabilities looked at from an ecosystem perspective, do not neutralise previous studies made on the complexity of the digital business strategy^17^ and the complexity of ecosystems,^19^ but rather add further dimensions to them, particularly bringing new insights into the capabilities needed when building an agenda in between the agents in future digital health in ecosystems dealing with non-communicable disease prevention and management under the ongoing economic crises and beyond. We believe that our results will advance the literature on digital health and health with a particular focus on the complexity perspectives. In addition, we trust that our findings will offer new conceptual insights to health stakeholders within industries and healthcare organisations to address the increasing demand for optimal and concept-driven solutions.

## Limitations and future work

Our study is not exempt from limitations. A part of the limitations is related to the nature of this study as a qualitative study. Despite a large amount of data and insights collected from different experts in the field, the study is still mainly concerned with the digitalisation of healthcare in a single, technologically advanced cultural context. Design of qualitative approach and thematic analysis were selected for this study because they allowed us to address the research gap directly, giving us future-oriented information about novel, not researched, key capabilities needed in future digital health innovation ecosystems focusing specifically on non-communicable diseases. The qualitative analysis method allowed the data to be collected directly from those front-line experts who are working on the phenomenon under investigation in several non-communicable disease areas such as stroke, mental, musculoskeletal disorders, Alzheimer’s, diabetes, etc. A more holistic focus on ecosystems and several non-communicable disease areas also leads us to future research directions. If patients become active participants in future health ecosystems, their expectations for personal health data and its usage should also be considered. This and wellness data also provide potential avenues for quantitative studies. In this vein, exploring the intersection of complex systems and open innovation could increase our understanding of the digitalisation of healthcare, both about ecosystems at the local and systemic levels.^54–56^ The longitudinal implications of health crises, such as the recent COVID-19 pandemic on healthcare systems and their digitalisation, remain to be explored further.

The purpose of this study was to explore what future capabilities are needed in digital health from an ecosystem perspective. The paper aimed to answer the research gap of future capabilities more holistically, looking at the interdependencies between the ecosystem actors in the area of multiple non-communicable diseases. However, there is a need for more deeper investigation of the meaning of each of the identified capabilities, from the perspective of each ecosystem actor and related to each non-communicable disease. The identified capabilities are also very setting- and health-system-dependent. Therefore, further investigation of the context-specific characters of orchestration could be an exciting area for future research.

## Conclusions

This qualitative interview-based study was designed to apply the complex systems theory for exploring the digital health phenomenon and essential required capabilities to shape the research agenda for next-generation health systems. This is important to address the healthcare requirements for rapidly ageing societies and chronic diseases. Eleven required capabilities were identified for digital health ecosystems to make those suitable for dealing with these demands. The major themes identified were focus on self-care, preventive healthcare, the use of non-medical and heterogeneous data to support better treatments, and clinical decisions. Innovative processes and actions were also found essential to push digital health forward. Our study provides the focus areas for digital health services to address the upcoming demands in healthcare ecosystems, including handling chronic diseases under normal and health crisis conditions.

## APPENDIX 1. Interview questionnaire of digital health interviews

1. Who are you, and what is your occupation?
2. What is digital health, in your opinion?
  - How would you define the term?
    - Why have you ended up at this conclusion?
    - What else could it be? Why?
3. How is it related to your own interest and work area?
  1. - Why?
4. Who will benefit from digital health research or activities?
  1. - How?
5. What are the most important areas to focus on concerning digital health research, in your opinion?
  1. - Why?
6. What is going to happen in the area of digital health in the coming 5 years?
  1. - How about the coming ten years?
    - Why do you think so?
7. What is speeding up this development?
8. What is driving this development, in your opinion?
9. How could we collaborate in the area of digital health in the future?
  1. - Can you identify potential and interesting areas for funding proposals, articles, or other collaboration, (if so what)?
